# BioBarcode: a general DNA barcoding database and server platform for Asian biodiversity resources

**DOI:** 10.1186/1471-2164-10-S3-S8

**Published:** 2009-12-03

**Authors:** Jeongheui Lim, Sang-Yoon Kim, Sungmin Kim, Hae-Seok Eo, Chang-Bae Kim, Woon Kee Paek, Won Kim, Jong Bhak

**Affiliations:** 1 Korean BioInformation Center, Korea Research Institute of Bioscience and Biotechnology, Daejeon, 305-806, Korea; 2 School of Biological Science, Seoul National University, Seoul, 151-742, Korea; 3 School of Computational Sciences, Korea Institute for Advanced Study, Seoul, 130-722, Korea; 4 Department of Life Science, Sangmyung University, Seoul, 110-743, Korea; 5 Division of Natural History, National Science Museum, Daejeon, 305-705, Korea

## Abstract

**Background:**

DNA barcoding provides a rapid, accurate, and standardized method for species-level identification using short DNA sequences. Such a standardized identification method is useful for mapping all the species on Earth, particularly when DNA sequencing technology is cheaply available. There are many nations in Asia with many biodiversity resources that need to be mapped and registered in databases.

**Results:**

We have built a general DNA barcode data processing system, BioBarcode, with open source software - which is a general purpose database and server. It uses mySQL RDBMS 5.0, BLAST2, and Apache httpd server. An exemplary database of BioBarcode has around 11,300 specimen entries (including GenBank data) and registers the biological species to map their genetic relationships. The BioBarcode database contains a chromatogram viewer which improves the performance in DNA sequence analyses.

**Conclusion:**

Asia has a very high degree of biodiversity and the BioBarcode database server system aims to provide an efficient bioinformatics protocol that can be freely used by Asian researchers and research organizations interested in DNA barcoding. The BioBarcode promotes the rapid acquisition of biological species DNA sequence data that meet global standards by providing specialized services, and provides useful tools that will make barcoding cheaper and faster in the biodiversity community such as standardization, depository, management, and analysis of DNA barcode data. The system can be downloaded upon request, and an exemplary server has been constructed with which to build an Asian biodiversity system http://www.asianbarcode.org.

## Background

DNA barcoding is the standardized minimal approach to facilitate biodiversity studies that include species identification and discovery. It helps researchers to understand evolutionary and genetic relationships by assembling molecular, morphological, and distributional data [1]. Species-level identification through DNA barcoding is usually accomplished by the retrieval of a short DNA sequence from a standard part of the genome (i.e., 650-base fragment of the 5' end of the mitochondrial cytochrome *c *oxidase I (COI) gene for animal species) from the specimen under investigation [2]. The barcode sequence from each unknown specimen is then compared with a library of reference barcode sequences derived from individuals of known identity [3].

The Consortium for the Barcode of Life (CBOL), which was launched in May 2004 and now includes more than 170 member organizations from 50 countries, is promoting DNA barcoding *sensu stricto *as the global standard for biological identification [4]. In contrast with the limited supply of taxonomic expertise, the need to assign specimens to known species arises every day and everywhere. Using molecular biomarkers, nonspecialists can assign specimens to known species - even specimens that can confound specialists (e.g., eggs, larvae, incomplete adults). Barcoding can therefore free taxonomists from the routine identification task of documenting new species. Next-generation DNA sequencing systems [5,6] will enable the rapid production of barcodes, thus eventually promoting the assignment of unknown individuals to classified species.

DNA barcoding *sensu lato *have reached out actively to new research areas other than taxonomy such as forensic science [7,8], the biotechnology and food industries, and animal diet [9,10]. Ecologists, environmental scientists, agricultural inspectors, public health officials, and other potential users with the need to identify specimens are exploring barcoding as a new approach to applied problems [11]. Taxon identification with diagnostic single-nucleotide polymorphisms (SNPs) and biodiversity assessment from environmental samples (e.g., soil and water) can also be considered DNA barcoding *sensu lato *[12].

The DNA barcoding pilot projects contain several large groups of animals such as birds [13], fish [14], cowries [15], spiders [16], amphibians [17], and several arrays of Lepidoptera [18-20]. In addition, DNA barcoding systems are now being established for other groups of organisms, including plants [21], macroalgae [22], fungi [23], protists [24], and bacteria [25]. The barcoding projects of Korea Barcode of Life (KBOL), launched in April of 2007, are currently collecting barcode data of vertebrates [26], invertebrates, land tracheophytes, and lower plants.

We built a DNA barcoding database and web server system, BioBarcode, which was developed as a part of KBOL project, to provide a reusable barcode construction system for more specific projects. In other words, BioBarcode is a bioinformatics template or platform rather than a specific DNA barcode server. The purpose of BioBarcode is to be used by biologists who have specific species information and want to build a DNA barcode database and server. It supports the compilation, storage, analysis, and publication of high-quality DNA barcode records. For many experimental biologists, building a local DNA barcoding system is expensive and time consuming. Therefore, BioBarcode will be useful for providing the tools needed to launch successful barcoding projects in the Asian biodiversity research community, including software for data management and analysis, data standards, and a data repository. To establish data standard, we have adopted the guidelines from CBOL and GenBank at the National Center for Biotechnology Information (NCBI) that must be satisfied for records to gain formal barcode status. Furthermore, it can be used for promoting international collaboration for building an Asian biodiversity system aiming to be the Asian biodiversity database server. Here, we introduce an exemplary web system using BioBarcode.

## Methods

### System architecture and scheme

**Figure 1:**
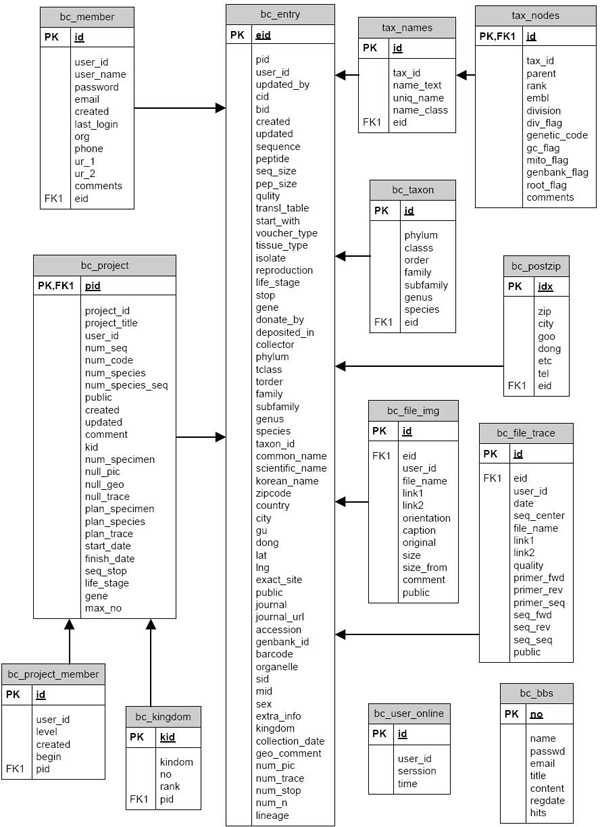
**BioBarcode system structure**. The BioBarcode database consists of 12 tables and implemented using mySQL RDBMS 5.0 as DBMS. The tables of image and trace files were separated to speed up and manage attachments easily.

The database structure of BioBarcode system consists of 12 tables as shown in Figure 1. These tables are mainly bc_entry (a table for management of general information on specimens), and following taxonomy, project, location, attached files (image and chromatogram), and a member management table (Table 1). We used a lineage table as tax_names and tax_nodes from the NCBI taxonomy database to search and navigate lineage. This table contains taxonomy information of 370,000 species and their lineages from kingdom to subspecies, therefore, users can choose taxonomy and lineage information by keyword suggestion. The tables of image and trace files, such as bc_file_img and bc_file_trace, were separated to speed up and manage attachments easily. Minimal information is required for member registration, and we accept various types of geographical information such as addresses, latitude and longitude positions on Google map, and zipcodes (this zipcode data is only available in South Korea). These are implemented using mySQL RDBMS 5.0 as DBMS.

**Table 1:** Tables used in the BioBarcode database

Table Name	Function
bc_taxon	Taxonomy information
tax_names	Taxonomy Name
tax_nodes	Taxonomy Node
bc_project_manager	Project Management
bc_kingdom	Project Classification
bc_project	Project Information
bc_postzip	Postal Address and ZIP code (geographical information)
bc_entry	Entry (general information of specimens)
bc_file_trace	Attached chromatogram file
bc_file_img	Attached Image File
bc_member	Registered Member Management
bc_bbs	Bulletin Board System for News

The BioBarcode database tables are mainly bc_entry (general information of specimens), and following taxonomy, project, location, attached files (images, chromatogram) and a member management table.

### Data uploads and repository

Anyone can create a project(s) by registering as a BioiBarcode user through the completion of a short online form http://www.asianbarcode.org/register.php. While the data upload of the Barcode of Life Data Systems (BOLD, http://www.barcodinglife.org) is carried out in two parts of specimen and sequence, BioBarcode system can be uploaded data in three parts: specimen, taxonomy, and sequence.

**Figure 2:**
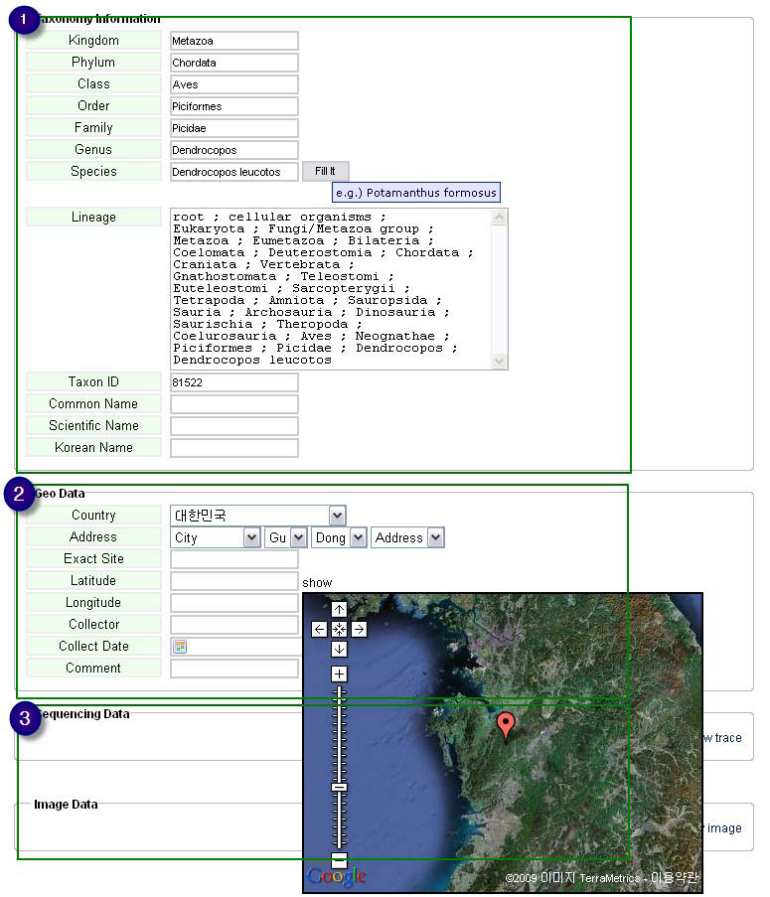
**Screenshot of the data entry for taxonomy and geographical distribution**. Clicking an icon using the Google map API makes an automatic entry of longitude and latitude of the selected location in geographical distribution. 1, Taxonomy Information; 2, Geographical Information; 3, Image and Chromatogram Files.

Key specimen data, such as voucher information and collection data with geographical distributions, is recorded in the specimen entry. Clicking an icon using the Google map API makes an automatic entry of longitude and latitude of the selected location in geographical information (Figure 2). In the taxonomy entry, partial information on a species, such as full taxonomy, lineage, taxon ID (linked with NCBI Taxonomy database), common name, scientific name, and Korean name, is input. In the sequence entry, the primary barcode information is DNA which is automatically translated to protein when the starting button is selected on DNA sequences. In the case of a barcode has already been published in journals, users can simply input it in the entry of literature information.

### Data collection and validation

BioBarcode provides users with a pathway for the direct submission of their data, which can include the information related to specimens, sequences, trace files, and images. Once data are submitted, if the information needs updating, an edit function is directly accessible from the sequence and specimen pages. Projects created by any registered user (a 'project manager' status will be acquired through the project creation) will be subject to optional security measurement, and all data records will remain private to a single researcher or to a group of collaborators until they opt for public release.

**Figure 3:**
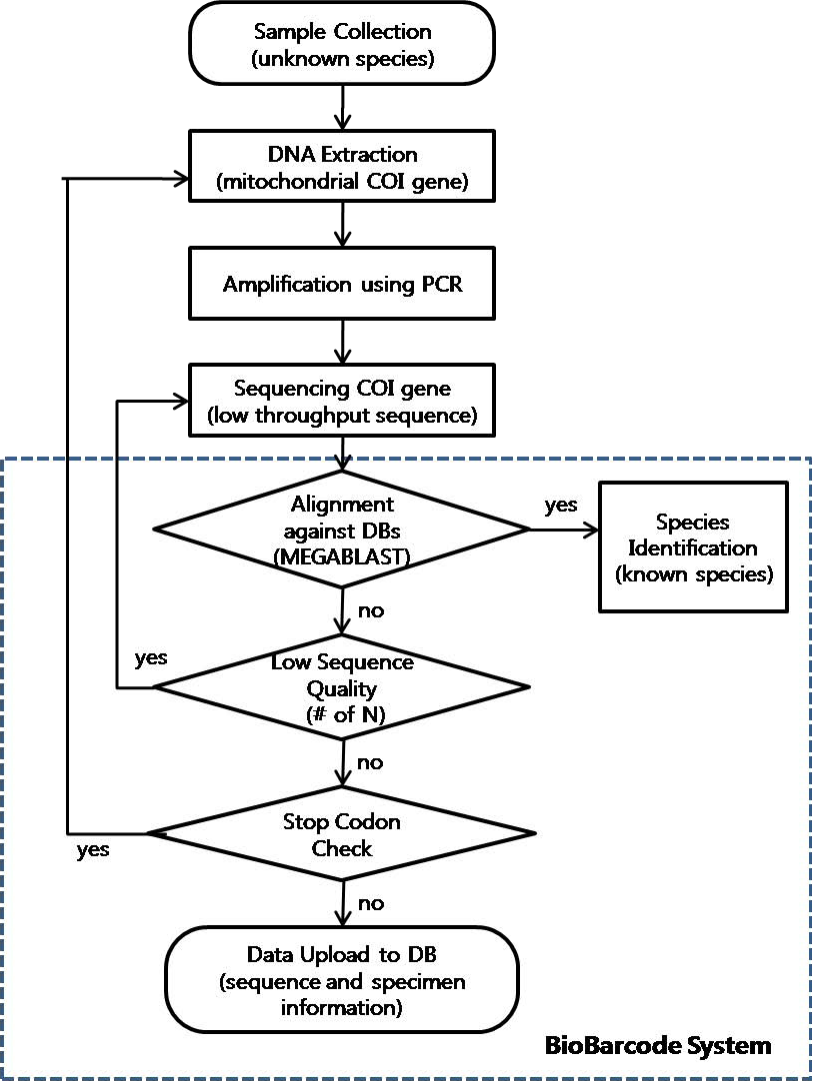
**Flowchart of BioBarcode DNA sequence identification and quality validation**. The flowchart is to explain the whole procedure from sample collection to data quality checking. The dotted line indicates how the processing occurs in BioBarcode system.

**Figure 4:**
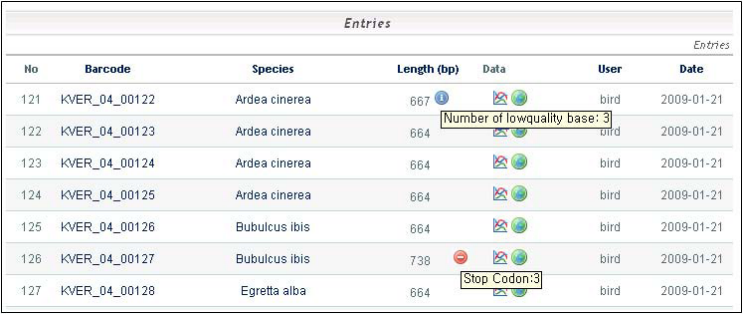
**Screenshot of the result page showing low quality sequences and stop codons**. The blue icon shows the presence of low quality bases in a sequence. Also, the red icon appears when the automatically translated amino acid sequence contains stop codons.

BioBarcode employs a function to assess data quality (Figure 3). All submitted DNA sequences which have gone through experimental steps such as sample collection, DNA extraction, amplification, and sequencing, are translated into proteins and then compared against already known COI proteins to verify that they are true COIs using MEGABLAST program with parameters of -p 95 -m 8 -e < > -b < >. The main target database is GenBank and any in-house database can be used to be compared with the input sequence. Sequences that pass this check are then examined for stop codons (to detect the presence of pseudo-genes). If any potential errors are detected, the sequence is flagged (Figure 4). Based on these results, the blue icon shows the presence of low quality bases. Similarly, the red icon appears when the automatically translated amino acid sequence contains stop codons. The final step is uploading the sequence data into the database with specimen information.

## Results and discussion

### Data access and searches

**Figure 5:**
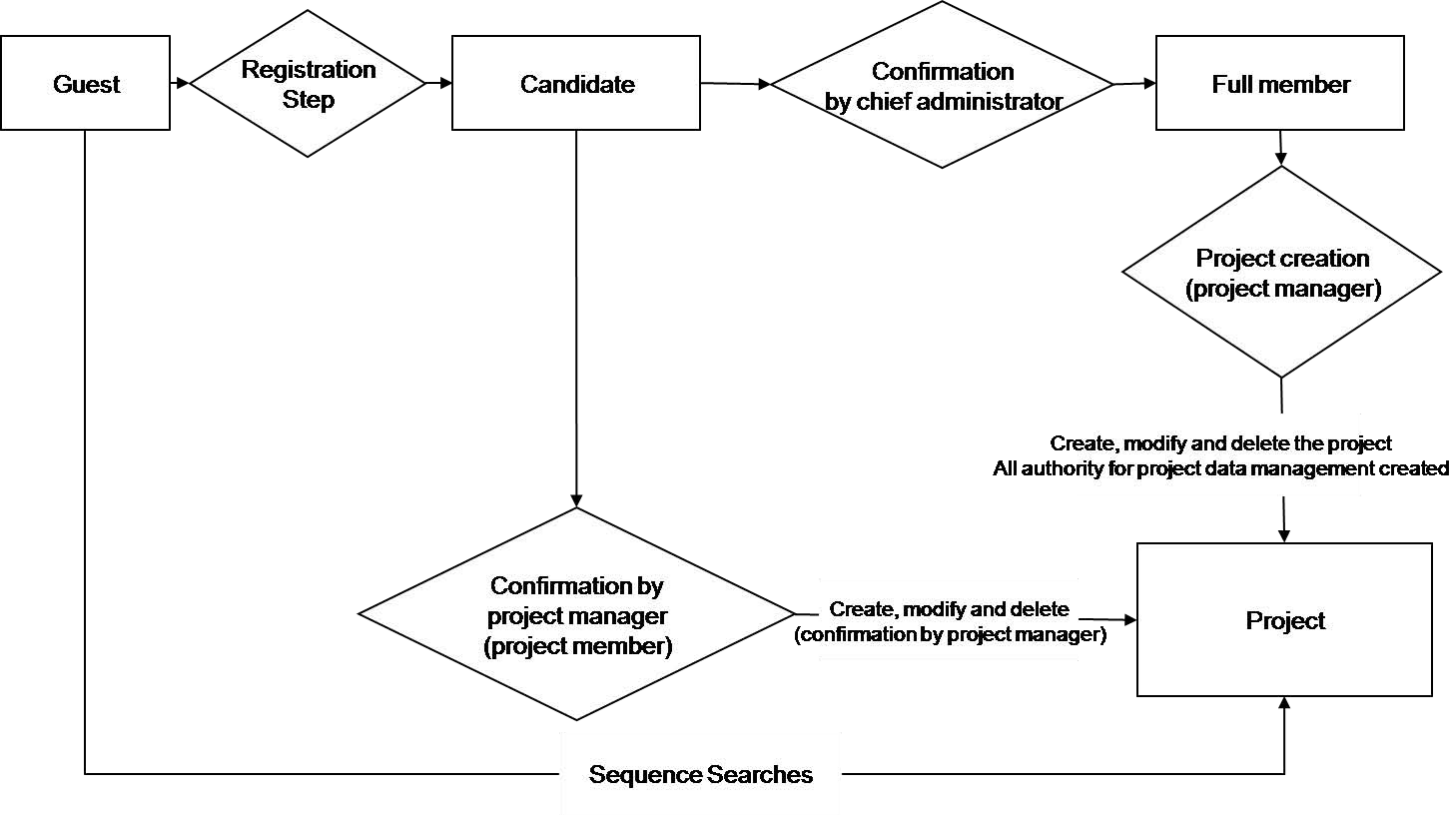
**Data access authority and roles based on membership**. The input and modification of barcode data depend on the level of members defined as chief administrator, project manager, full member, and guest member. Chief administrator, project manager, and project members only can input the barcode data. And the data retrieval is restricted to project members and the chief administrator if the project is not open to the public.

The data access authority in BioBarcode system is based on membership level. The barcode data input and modification depend on the member level, defined as chief administrator, project manager, full member, and guest member. Any project data is accessible only when a user is registered to the project and certified by the project manager. However, up to two items, such as specimen image and species/specimen name, can be accessed by any unregistered member. Also, barcode data input is available for chief administrator, project manager, and project members. Data retrieval is restricted to project members and the chief administrator if the project is not open to the public. Full members can create and manage a project (Figure 5).

A data search can be performed by the input of a DNA sequence or a keyword. Sequence-based data retrieval is done by creating a database containing publically available mitochondrial COI gene data registered in GenBank and Korean barcode projects. The search of an input sequence data is accomplished by using the MEGABLAST program. The search results are shown in the order of sequence similarity, sequence length, and gap opening. Users can filter the results by E-value (default is 10E-5). On the same search web page, there is the keyword-based data retrieval section. Users can choose an open or a closed project type, and the barcode ID, sample ID, collector, and scientific name can be then used as a search parameter. For a historical reason of providing the service in Korea, a Korean name can be used as a search parameter. The results from both methods are directly linked to the entry of the general information of specimens.

### User interface

**Figure 6:**
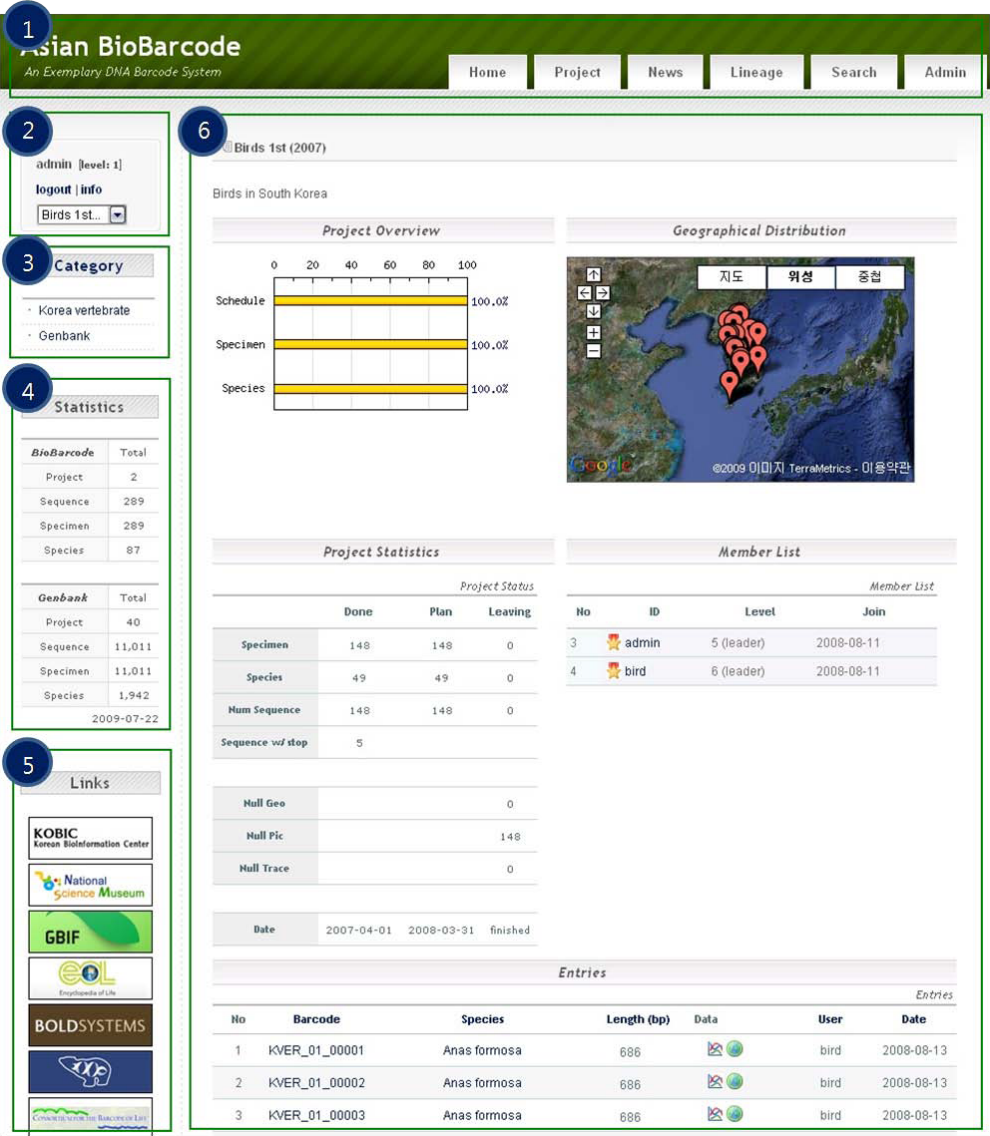
**BioBarcode user interface**. Users can search the BioBarcode DB using five entries: 1, main menu bar; 2, log-in system; 3, category; 4, statistics; 5, other links. The project page (6 of Fig. 6) shows six sections such as project title, overview, geographical distribution, statistics, member list, and entries.

As shown in Figure 6, the BioBarcode database consists of five entries: main menu bar, log-in system, category, statistics, and other links to related organizations to facilitate effective and user-friendly database management.

Through the 'Lineage' page of the main menu section (1 of Figure 6), taxonomy information based on input data which requires authorization can be identified. Personal information and registered project lists are available in the login state (2 of Figure 6). The 'Category' registered by administrator is linked with the 'Project' (3 of Figure 6). The statistics of registered species, specimen, sequence, projects, and trace files can be viewed on the 'Statistics' page (4 of Figure 6). The 'Project' page (6 of Figure 6) consists of six sections: project title, general overview (e.g., number of species, specimens and sequencing reaction, dates of start and end project,), geographical distribution, statistics, member list, and entries (e.g., barcode ID, species, and sequence with trace data).

Recently mobile interface program have become available such as Yahoo's blueprint interface http://mobile.yahoo.com/developers. We plan to implement the mobile application interface so that researchers can easily and rapidly deposit and monitor data in real time for sampling locations, collections, and observations. Another major issue is various Asian languages supports by the BioBarcode DB/Server construction system. We will include language pack in the next version of BioBarcode.

### Computational resources

The BioBarcode system was developed with open source software. PHP and JavaScript were used for most web pages and the main system so that any future developers can join or develop their own based on BioBarcode. mySQL was used for database management and MEGABLAST was used to search sequence identity. BioBarcode does not require much computing resources, and a stand-alone PC workstation is enough to run the server. The BioBarcode server is currently optimized for IE7 internet browsers. Therefore, some javascript or ajax functions may not work correctly with other internet browsers. Internet standards will be kept more strictly in the next versions.

## Conclusion

A DNA barcode server and database construction system, BioBarcode, and an exemplary server are presented, aiming to provide a platform for biological researchers who want to establish their own DNA barcode database and web server system compatible with international standards that meet the criteria in the International Nucleotide Sequence Database Collaboration (INSDC, which includes GenBank, the European Molecular Biology Laboratory, and the DNA Data Bank of Japan). BioBarcode is targeted to Asian researchers who have many local biodiversity resources. It intends to be easy to run and maintain with inexpensive open source software.

## Competing interests

The authors declare that they have no competing interests.

## Authors' contributions

JB directed the study and helped draft the manuscript. WK coordinated the collaboration and the study associated with BioBarcode and KBOL. CBK and WKP were involved in reviewing and critically revising it for intellectual content. SYK, SMK and HSE provided technical assistance while constructing the database. JHL conceived the study and wrote the manuscript. All authors read and approved the final manuscript.

## Note

Other papers from the meeting have been published as part of *BMC Bioinformatics* Volume 10 Supplement 15, 2009: Eighth International Conference on Bioinformatics (InCoB2009): Bioinformatics, available online at http://www.biomedcentral.com/1471-2105/10?issue=S15.
